# Reduced Susceptibility to Extended-Spectrum β-Lactams in *Vibrio cholerae* Isolated in Bangladesh

**DOI:** 10.3389/fpubh.2016.00231

**Published:** 2016-10-18

**Authors:** Daniela Ceccarelli, Munirul Alam, Anwar Huq, Rita R. Colwell

**Affiliations:** ^1^Maryland Pathogen Research Institute, University of Maryland, College Park, MD, USA; ^2^International Center for Diarrheal Disease Research, Bangladesh (icddr,b), Dhaka, Bangladesh; ^3^Maryland Institute for Applied Environmental Health, University of Maryland, College Park, MD, USA; ^4^Center for Bioinformatics and Computational Biology, University of Maryland, College Park, MD, USA; ^5^Johns Hopkins Bloomberg School of Public Health, Johns Hopkins University, Baltimore, MD, USA

**Keywords:** *Vibrio cholerae*, cholera, Bangladesh, extended-spectrum β-lactams, carbapenems, antibiotic resistance, aquatic environment, clinical environment

## Abstract

β-lactams are antibiotic molecules able to inhibit cell wall biosynthesis. Among other mechanisms, resistance in Gram-negative bacteria is mostly associated with production of β-lactamase enzymes able to bind and hydrolyze the β-lactam ring. Extended-spectrum β-lactamases extend this ability also to third- and fourth-generation cephalosporins, as well as to carbapenems and monobactams. *Vibrio cholerae* is the causative agent of epidemic cholera and a public health burden for developing countries like Bangladesh. Although appropriate oral or intravenous rehydration is the therapy of choice for cholera, severe infections and *V. cholerae*-associated septicemia are treated with antimicrobial drugs, including doxycycline, erythromycin, azithromycin, ciprofloxacin, and/or third-generation cephalosporins. In the years after the introduction of antibiotics in clinical practice, *V. cholerae* developed resistance to commonly used drugs worldwide mostly through gene acquisition *via* horizontal gene transfer. Reduced susceptibility of *V. cholerae* to third-generation cephalosporins has been occasionally documented. However, carbapenemase-producing *V. cholerae* has been reported at higher rates than resistance to extended-spectrum β-lactams, mainly associated with *bla*_NDM-1_ emergence and successful plasmid dissemination. Recent findings suggest limited β-lactam resistance is present in *V. cholerae* O1 isolates collected during ecological and epidemiological surveillance in Bangladesh. However, a trend to intermediate-susceptibility insurgence was observed. Horizontal gene transfer of β-lactam resistance from enteric pathogens to environmental microorganisms should not be underrated, given the ability of *V. cholerae* to acquire new genetic information.

## Introduction

β-lactams are assorted antibiotic molecules able to inhibit cell wall biosynthesis. Resistance to these drugs can be the result of altered permeability, antibiotic target site alteration, or antibiotic degradation (1). The latter represents the primary resistance mechanism in Gram-negative bacteria producing β-lactamase enzymes able to bind and hydrolyze the β-lactam ring (2). β-lactamases have extensively diversified in response to the clinical use of new generations of β-lactams, including the clinically significant extended-spectrum β-lactamases (ESBLs) CTX-M-, TEM-, and SHV-type enzymes (3). Carbapenemases are ESBLs that recognize almost all hydrolyzable β-lactams, including carbapenems (imipenem, ertapenem, meropenem, and doripenem), the last-line therapeutics to treat multidrug-resistant Gram-negative infections (4). Carbapenem resistance in *Enterobacteriaceae* constitutes an important and growing public health threat, especially since the appearance of the powerful enzyme NDM-1 (5), whose presence during the past years was reported worldwide (6).

The majority of diarrheal diseases in Bangladesh are endemic and waterborne since surface water can be heavily contaminated due to poor sanitation and hygiene (7) and access to safe drinking water is problematic (8). Together with enterotoxigenic *Escherichia coli, Vibrio cholerae* is one of the leading causes of enteric infections in the country. *V. cholerae* is a natural inhabitant of estuarine brackish waters and it can thrive in the human gut, causing mild to severe infections and cholera. Today, more than 200 serotypes of *V. cholerae* have been documented, with O1 and O139 being the only serotypes associated with epidemic cholera (9). Cholera can occur both as endemic disease with seasonal peaks and in epidemics associated with floods, droughts, and cyclones that occur in the country (10). Infections other than cholera are caused by non-epidemic *V. cholerae* serogroups, collectively referred to as *V. cholerae* non-O1/non-O139, with infections reported worldwide (11, 12).

According to World Health Organization guidelines, oral rehydration is the therapy of choice for *V. cholerae* infections, independent of serotype (13). It is recommended that severe infections and septicemia be treated with antimicrobial therapy, choosing an effective antibiotic according to local antibiotic susceptibility patterns. Doxycycline is recommended as the first-line treatment for *V. cholerae* O1 or O139 infections in adults, while erythromycin or azithromycin are recommended for children and pregnant women. Ciprofloxacin and/or third-generation cephalosporins (ceftazidime and ceftriaxone) are recommended for *V. cholerae* non-O1/non-O139 infections (14).

After the introduction of antibiotics into clinical practice, *V. cholerae* remained relatively susceptible till the end of the 1970s (15). Within a few years however, this scenario changed dramatically, with *V. cholerae* strains found to be resistant to commonly used drugs worldwide (16–18), a phenomenon likely attributable to indiscriminate use of antibiotics. Today, *V. cholerae* can be resistant virtually to all commonly used antibiotics, including ampicillin, quinolones, ciprofloxacin, tetracycline, cotrimoxazole, and macrolides (19). The reasons are multiple and rely on chromosomal mutations, enhanced efflux pumps, and acquisition of drug altering enzymes *via* horizontal gene transfer (20). The latter has proven to be the most powerful, as a result of a variety of mobile elements circulating amongst *V. cholerae*, such as conjugative plasmids (21, 22), integrative conjugative elements (23), and mobile genomic islands (24).

The full extent of antibiotic resistance in *V. cholerae* is not yet known because of limited data. Non-cholera *Vibrio* infections are not mandatorily notifiable in several countries and annual figures on cholera cases may be significantly under estimated, especially when labeled as “acute watery diarrhea,” in south-eastern and central Asia (10). The first clinical multi-resistant *V. cholerae* O1 in Bangladesh was isolated in 1979, displaying plasmid-mediated resistance to tetracycline, ampicillin, kanamycin, streptomycin, and trimethoprim–sulfamethoxazole (25). Several studies since and the current dissemination of carbapenemases and ESBLs make it mandatory to understand this phenomenon, especially because of the higher mortality, morbidity, and increased health treatment costs associated with resistance to β-lactams (26).

## Third-Generation Cephalosporin and Carbapenem Resistance in *V. cholerae*

The first reports of *V. cholerae* O1 showing strong reduced susceptibility to cefotaxime, ceftazidime, and/or aztreonam appeared during the first Argentinean cholera outbreak, which was caused by an ESBL-producing isolate in the 1990s (27). This phenotype was associated with two plasmid-mediated ESBLs of the CTX-M- and PER-2-type (28). After a long hiatus, new cases of reduced susceptibility to third-generation cephalosporins were described in *V. cholerae* O1, mostly in the Indian Subcontinent. Resistance to ceftriaxone was originally reported in pediatric cases from Puducherry, India in 2008–2010 (29, 30) and similar findings were described in Karnataka, South India, where cephalosporin-resistant strains were found to produce ESBLs (31). Due to lack of genetic analysis, the exact nature of the resistance mechanisms of the reduced susceptibilities is not clear. The most recent genetic characterization of multidrug-resistant, ESBL-producing *V. cholerae* was a plasmid-borne *bla*_TEM-63_ in *V. cholerae* O1 associated with a cholera outbreak in South Africa in 2008 (32), and an ISCR1-mediated *bla*_PER-1_ embedded in a class 1 integron on a conjugative IncA/C plasmid in a clinical *V. cholerae* non-O1/non-O139 isolate from human blood in China (33).

Clearly, extended-spectrum β-lactamases are uncommon in *V. cholerae*, as well as in other *Vibrionaceae*, with only two findings to date. *Vibrio fluvialis* isolated from cholera-like diarrheal patients in West Bengal, India in 2009 encoded a 150-kb plasmid harboring *bla*_SHV_ and *bla*_CTX-M-3_, together with the quinolone resistance gene *qnrA1*, and ciprofloxacin-resistance gene *aac(6)-Ib-cr* (34). *Vibrio parahaemolyticus* of food origin from China was reported to carry either the AmpC β-lactamase *bla*_CMY-2_ on a 150-kb IncA/C-type conjugative plasmid, previously described in *Enterobacteriaceae* (35), or a 200-kb conjugative plasmid encoding *bla*_PER-1_, conferring resistance to both third- and fourth-generation cephalosporins (36, 37).

Carbapenemase-producing *V. cholerae* has been reported at a higher rate than ESBLs. The first description in Western countries was in southern France, where *V. cholerae* non-O1/non-O139 was isolated from a yellow-legged gull and found to encode both *bla*_VIM-1_ and *bla*_VIM-4_ on an IncA/C plasmid (38). Shortly after, the novel transferable carbapenemase *bla*_VCC-1_ was identified in a non-toxigenic strain of *V. cholerae* non-O1/non-O139 during antimicrobial resistance surveillance of food in Canada (39). VCC-1, the first class A carbapenemase to be found in a member of the *Vibrionaceae*, can hydrolyze penicillin, first-generation cephalosporins, aztreonam, and carbapenems but not second- and third-generation cephalosporins. Most notably, *bla*_NDM-1_ has been detected in environmental *V. cholerae* non-O1/non-O139 in southern Vietnam (40), in clinical *V. cholerae* O1 in India together with the AmpC β-lactamase *bla*_DHA_ gene (41), in a polymicrobial infection (*V. cholerae, Acinetobacter baumannii, Staphylococcus aureus*, and *Pseudomonas aeruginosa*) in the UK (42), and from water seepage in New Delhi, India (43). The successful spread of *bla*_NDM-1_ can be attributed to its association with conjugative plasmids (44) and emphasizes the extent to which *bla*_NDM-1_ can disseminate among different species outside of the *Enterobacteriaceae*.

## ESBL- and Carbapenemase-Mediated Resistance in Bangladesh

Antibiotic resistance is a serious threat in Bangladesh and has been for decades, most likely a result of unrestricted use of antimicrobial drugs to treat enteric infections, particularly those caused by *V. cholerae, Salmonella, Shigella*, and enterotoxigenic *E. coli* (45). In the recent years, ESBL- and carbapenemase-mediated resistance has been found to be ubiquitous and has been detected in a variety of bacterial hosts.

*bla*_CTX-M-15_ is the dominant ESBL variant circulating in Bangladesh. It has been reported mainly in *E. coli* found in wild birds and aquatic systems (46), in household pigeons (47), poultry (48), urban surface water (49), and in epidemic *E. coli* isolates from both patients and crows scavenging poorly managed hospital waste dumps (50). On occasion, other ESBL genes have been found to be associated with *bla*_CTX-M-15_, such as *bla*_CTX-M-14_ in wild birds (51), or *bla*_CTX-M-27_, *bla*_SHV-2_, and/or *bla*_SHV-12_ in *E. coli* and *Enterobacter cloacae* from environmental urban water (52). The clinical scenario is not very different, with *bla*_CTX-M-15_ prevailing (53), although bacterial species other than *E. coli* with the ESBL phenotype have been described (54–58). The first clinical *Salmonella typhi* positive for both *bla*_TEM_ and *bla*_CTX-M_ was recently reported to have been isolated from diarrheal patients in Dhaka (59). Overall, dissemination of ESBLs in Bangladesh seems to have reached all ecological niches, indicating that environmental contamination by antibiotic resistance is already quite high and probably widespread, from coastlines of the Bay of Bengal, to urban Dhaka, and to rural inland areas.

Emergence of *bla*_NDM-1_-mediated carbapenemase resistance was first described in Bangladesh in the *Enterobacteriaceae* in 2010 (60). Subsequent retrospective studies demonstrated the presence of *bla*_NDM-1_ in clinical *Klebsiella pneumoniae* isolated in 2008 (61). The same investigators also reported a 9% prevalence of fecal carriage of plasmid-encoded *bla*_NDM-1_ in diverse *Enterobacteriaceae* in the patient population (62). The problematic spread of carbapenemase in Bangladesh has been documented with the isolation of clinical *A. baumannii, P. aeruginosa*, and *K. pneumoniae* carrying genes encoding multiple enzymes, i.e., *bla*_VIM-1_, *bla*_VIM-2_, *bla*_IMP-1_, *bla*_IMP-2_, and/or *bla*_NDM-1_ (63). The same bacterial species have been detected in environmental water/sewage samples collected in Dhaka (64), documenting a high level of environmental distribution of *bla*_NDM-1_, a worrisome finding given the high levels of sewage-derived bacteria routinely isolated from drinking water in Bangladesh (65).

The role of plasmids in the successful spread of β-lactamase genes has been extensively described (66, 67) and their involvement in antibiotic resistance epidemiology in Bangladesh is no different. Although limited data are available, conjugative plasmids of various sizes (20–100 MDa) have been reported to carry ESBL genes (53, 61, 68), *bla*_NDM-1_, and/or other carbapenemases (60, 63), mostly in the metropolitan area of Dhaka.

## Antimicrobial Resistance Surveillance in *V. cholerae* in Bangladesh

To date, ESBL or carbapenemase-producing *V. cholerae* in Bangladesh have not been reported. *V. cholerae, E. coli*, as well as other *Enterobacteriaceae*, can coexist in different ecological niches. Given the ability of conjugative plasmids to transfer naturally between enterobacterial populations in the intestinal gut (68), the aquatic environment is now an ideal setting for acquisition and dissemination of antibiotic resistance (69), and the horizontal transfer of ESBL/carbapenemase genes to *V. cholerae* cannot be excluded.

In this perspective, we investigated β-lactam resistance^1^ in *V. cholerae* O1 collected during ecological and epidemiological surveillance in Bangladesh, the sampling and isolation details of which are described elsewhere (70). A total of 460 *V. cholerae* O1 isolates were collected between 2009 and 2014 in the provinces of Mathbaria (MB; southwestern Bangladesh) and Chhatak (CH; northeastern Bangladesh) and analyzed. The set of strains included clinical (C) and environmental (E) isolates (MB: C = 178 and E = 120; CH: C = 141 and E = 21), either from fecal samples of cholera patients at local health-care facilities or from ponds used for drinking water and other domestic purposes.

**Figure 1:**
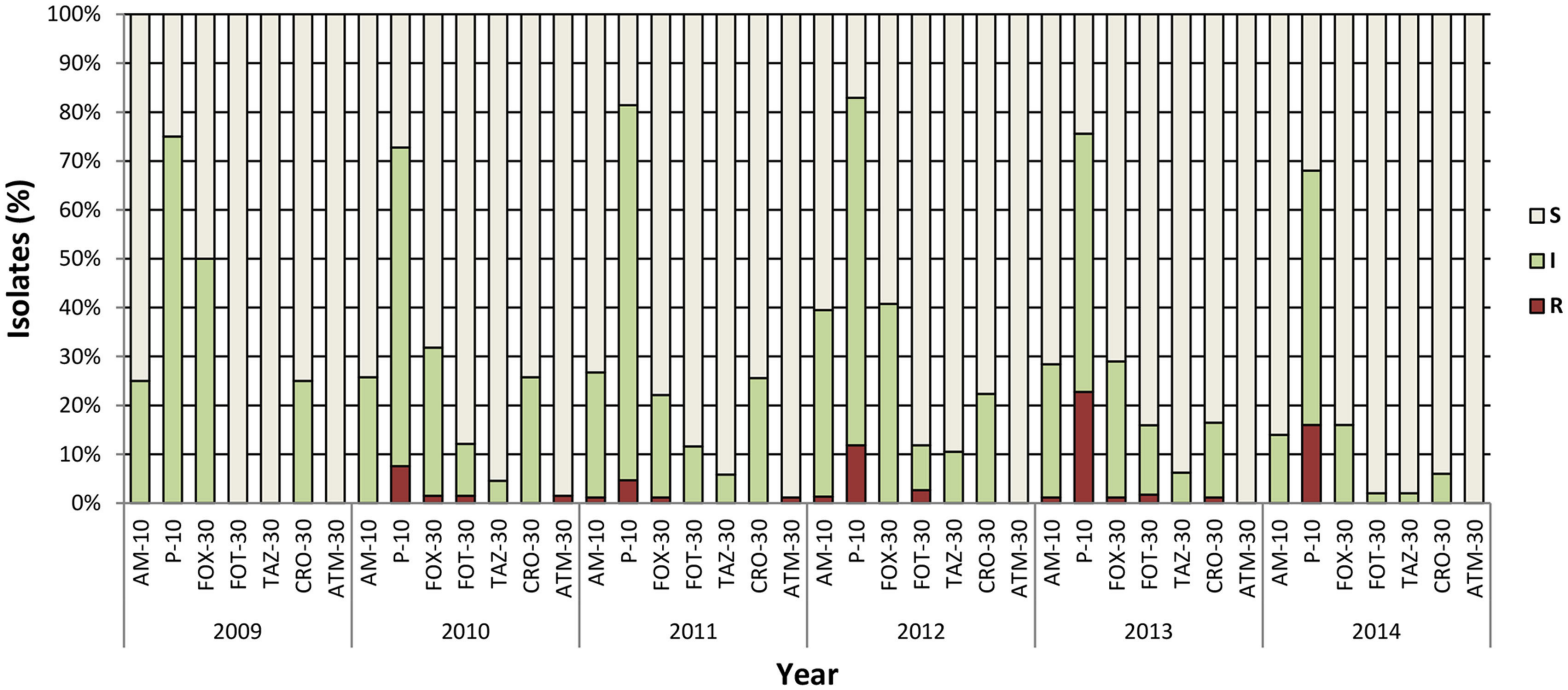
**Antibiotic sensitivity pattern of *V. cholerae* O1 during epidemiological surveillance (2009–2014)**. Number of isolates varied per year depending on results of sampling: 2009 (*n* = 4), 2010 (*n* = 66), 2011 (*n* = 86), 2012 (*n* = 76), 2013 (*n* = 176), and 2014 (*n* = 50). AM, ampicillin; P, penicillin; FOX, cefoxitin; FOT, cefotaxime; TAZ, ceftazidime; CRO, ceftriaxone; ATM, aztreonam. Concentrations are expressed as micrograms per disk. All isolates were susceptible to cefepime and imipenem. S, susceptible; I, intermediate; R, resistant.

Resistance to penicillin (ampicillin and penicillin), monobactams (aztreonam), carbapenems (imipenem), second- (cefoxitin), third- (cefotaxime, ceftazidime, and ceftriaxone), and fourth- (cefepime) generation cephalosporins was tested by disk diffusion, and the results were interpreted according to CLSI clinical breakpoints, for *V. cholerae* (71) or *Enterobacteriaceae* (72). All 460 *V. cholerae* isolates were found to be susceptible to imipenem and to fourth-generation cephalosporin cefepime. Seventy-two isolates showed resistance to one or more third-generation cephalosporins (cefoxitin, cefotaxime, and ceftazidime) ampicillin, and aztreonam (Table 1). Of those isolates, 57 were resistant only to penicillin. Intrinsic resistance to penicillin has been observed in several *V. cholerae* strains, including *V. cholerae* N16961 (73), and it is likely to be mediated by penicillin insensitive transglycolase domains in penicillin binding proteins PBP1A and PBP1B. The majority of isolates were also resistant to carbenicillin (data not shown), very likely correlated with intrinsic resistance to penicillin, as described previously in *V. parahaemolyticus* (74). The second most common resistance was to third-generation cephalosporin, cefotaxime alone, or in combination with penicillin, ceftriaxone, or aztreonam. The latter was found only in Mathbaria. Overall, no remarkable difference was observed between the two geographical locations (Table 1) or during the 5 years of the study (Figure 1). As expected, the majority of resistant isolates were of clinical origin, compared to environmental sources (Table 1), and the most extensive resistance profile was observed in a clinical strain isolated in 2013 from Mathbaria (Am, Pen, Fox, and Cro). Interestingly, several clinical isolates showed an intermediate phenotype, indicating evolution toward a resistant phenotype in the clinical environment, where the selective pressure of antibiotic use/misuse is higher than in the aquatic environment, where antibiotics may be less prevalent or at lower concentration.

**Table 1:** **Susceptibility vs. resistance (%) in *V. cholerae* O1 isolates from Bangladesh**.

Antibiotic^a^	Mathbaria (*n* = 298)						Chhatak (*n* = 162)					
	Clinical (*n* = 178)			Environmental (*n* = 120)			Clinical (*n* = 141)			Environmental (*n* = 21)		
	S	I	R	S	I	R	S	I	R	S	I	R
Ampicillin	63	35	2	73	26	1	80	20	0	90	10	0
Penicillin	16	67	17	23	63	14	35	52	13	19	76	5
Cefoxitin	62	36	2	77	22	1	77	23	0	81	19	0
Cefotaxime	82	17	1	87	12	1	95	3	2	100	0	0
Ceftazidime	93	7	0	92	8	0	96	4	0	100	0	0
Ceftriaxone	75	24	1	84	16	0	84	16	0	95	5	0
Aztreonam	84	15	1	89	10	1	88	12	0	95	5	0

*S, susceptible; I, intermediate; R, resistant*.

*^a^All isolates were susceptible to cefepime and imipenem*.

Isolates showing reduced susceptibility were screened to detect the ESBL (*bla_CTX_, bla_TEM_*, and *bla_SHV_*), AmpC (*bla_MOX_, bla_CIT_, bla_DHA_, bla_ACC_, bla_EBC_*, and *bla_FOX_*), and carbapenemase genes (*bla_IMP_, bla_SPM_, bla_VIM_, bla_BIC_, bla_NDM_, bla_KPC_, bla_AIM_, bla_SIM_, bla_DIM_*, and *bla_GIM_*), as previously reported (75–79). The results were negative for all isolates. The combination disk test results with clavulanic acid to detect ESBL for cefotaxime and ceftazidime-resistant isolates (72) and the phenotypic AmpC disk test with Tris–EDTA (80) were also negative, confirming the absence of an ESBL/AmpC phenotype. To date, the enzyme(s) responsible for the reduced susceptibility phenotype have not been identified. The presence of alternative resistance mechanisms or an intrinsic resistance cannot be ruled out, as observed earlier for *V. cholerae* non-O1/non-O139 in Germany (81) and for *V. parahaemolyticus* isolates from shellfish in Italy (82).

## Concluding Remarks

*Vibrio cholerae* remains quite susceptible to β-lactams, despite the fact that other enteric pathogens, mostly *Enterobacteriaceae*, have developed this resistance in the same geographic regions (52, 59, 63). Yet, a trend showing an increase in intermediate-susceptible isolates was observed, especially in clinical settings, highlighting a developmental path to a resistance phenotype.

The contribution of conjugative plasmids, or other mobile elements, in the horizontal acquisition of genetic factors conferring β-lactam resistance must not be underestimated. It has been established that *V. cholerae*, independent of serotype, has a plastic genome and a long history of successful association with plasmids that have helped to shape the multi-resistant phenotype that now characterizes this pathogen (18, 22, 24). Plasmids encoding ESBLs have been shown to possess a wide bacterial host range (67) and the ICEs of the SXT/R391 family play a major role in antibiotic resistance acquisition by the *Vibrionaceae* (83). ICE*Pmi*Jpn1, encoding *bla*_CMY-2_ and conferring resistance to third-generation cephalosporins (77), can successfully be transferred among clinically relevant *Enterobacteriaceae* and readily disseminated to *V. cholerae*, one of its natural bacterial hosts. *V. cholerae* may very well act as an environmental reservoir for antibiotic resistance genes, contributing to genetic plasticity and dissemination. Finally, the presence of β-lactamase-producing bacteria in the aquatic environment renders environmental surveillance mandatory in order to monitor the role of the natural environment in the distribution of antibiotic resistance and to track potentially clinically relevant *V. cholerae* isolates.

## Author Contributions

The work was conceived and performed by DC. All authors discussed, read, contributed to, and approved the final manuscript.

## Conflict of Interest Statement

The authors declare that the research was conducted in the absence of any commercial or financial relationships that could be construed as a potential conflict of interest.

## Footnotes

^1^Routine antibiotic resistance screening was also performed, results of which are beyond the scope of this article and will be reported elsewhere.
